# Cranial ultrasound screening in late preterm infants

**DOI:** 10.1186/1824-7288-40-S2-A20

**Published:** 2014-10-09

**Authors:** M Fumagalli, LA Ramenghi, A De Carli, L Bassi, F Dessimone, S Pisoni, M Groppo, A Ometto, I Sirgiovanni, F Mosca

**Affiliations:** 1 NICU, Department of Clinical Sciences and Community Health, Fondazione IRCCS Cà Granda Ospedale Maggiore Policlinico, Università degli Studi di Milano, Milan, Italy; 2 Neonatal Intensive Care Unit, Istituto Giannina Gaslini, Genoa, Italy

## 

Late preterm births have enormously increased in the last decades and there is mounting evidence showing that infants born late preterm are less healthy than infants born at term [1] and they are more likely to develop neonatal morbidities (temperature instability, respiratory distress syndrome, excessive weight loss and dehydration requiring intravenous infusion, sepsis, hypoglycemia and jaundice requiring phototherapy) [2].

More recently, an increased neuromorbidity has been documented and long-term neurodevelopmental impairments (poor school performance, early intervention services, special education needs) have been reported in this population [3,4]. The neuromorbidity of the late preterm infants has been attributed to both the potential detrimental neurological effects (extrinsic vulnerability) of the morbidities these babies experience in the neonatal period, and to the intrinsic brain vulnerability. Advances in neuroimaging techniques have highlighted a higher intrinsic vulnerability of the late preterm brain due to the structural and molecular immaturity of the developing brain at specific gestational ages [5,6].

Therefore, late preterm infants have a risk to develop brain lesions which is lower than more premature babies but higher than term newborns and they can be affected by brain lesions common to both preterm and term infants [7]. However, the incidence of brain abnormalities in this specific population has never been investigated as late preterm infants have long been considered a large and low-risk population.

Considering that most of the brain lesions are clinically subtle or silent during the neonatal period, a cranial ultrasound screening may play a role in: 1. detecting babies at risk of impaired neurodevelopment later in childhood and who may benefit from early intervention programs; 2. identifying the most significant perinatal risk factors associated with brain abnormalities in such a large low-risk population in order to target the potential need for cranial ultrasound at birth. Based on these assumptions we performed a cranial ultrasound screening project on late preterm infants. Our preliminary data (unpublished data) suggest that lower gestational age, within the late preterm period, and early neonatal morbidities, can provide an indication at birth to undergo a cranial ultrasound scan as they are associated with a higher risk to develop brain abnormalities. Late preterm infants represent a vulnerable population and investigation and follow-up program should be modulated according to the prenatal, perinatal and postnatal characteristics.

Follow-up studies are needed to correlate neonatal ultrasound findings with long-term neurobehavioral outcomes in late preterm infants.
